# Role of Mecp2 in Experience-Dependent Epigenetic Programming

**DOI:** 10.3390/genes6010060

**Published:** 2015-03-06

**Authors:** Christoph A. Zimmermann, Anke Hoffmann, Florian Raabe, Dietmar Spengler

**Affiliations:** Max Planck Institute of Psychiatry, Translational Research, Kraepelinstr. 2-10, Munich 80804, Germany; E-Mails: christoph_zimmermann@psych.mpg.de (C.A.Z.); hoffmann@psych.mpg.de (A.H.); florian_raabe@psych.mpg.de (F.R.)

**Keywords:** early-life stress, Mecp2, HPA axis, epigenetic programming, *Avp*, *Crh*, *Pomc*

## Abstract

Mutations in the X-linked gene *MECP2*, the founding member of a family of proteins recognizing and binding to methylated DNA, are the genetic cause of a devastating neurodevelopmental disorder in humans, called Rett syndrome. Available evidence suggests that MECP2 protein has a critical role in activity-dependent neuronal plasticity and transcription during brain development. Moreover, recent studies in mice show that various posttranslational modifications, notably phosphorylation, regulate Mecp2’s functions in learning and memory, drug addiction, depression-like behavior, and the response to antidepressant treatment. The hypothalamic-pituitary-adrenal (HPA) axis drives the stress response and its deregulation increases the risk for a variety of mental disorders. Early-life stress (ELS) typically results in sustained HPA-axis deregulation and is a major risk factor for stress related diseases, in particular major depression. Interestingly, Mecp2 protein has been shown to contribute to ELS-dependent epigenetic programming of *Crh*, *Avp*, and *Pomc*, all of these genes enhance HPA-axis activity. Hereby ELS regulates Mecp2 phosphorylation, DNA binding, and transcriptional activities in a tissue-specific and temporospatial manner. Overall, these findings suggest MECP2 proteins are so far underestimated and have a more dynamic role in the mediation of the gene-environment dialog and epigenetic programming of the neuroendocrine stress system in health and disease.

## 1. Introduction

The field of epigenetics has developed from different research lines that continue to shape our current understanding. As far back as the forties, Conrad Waddington referred to epigenetics as the study of epigenesis; that is the question of how genotypes can give rise to different phenotypes during development [1]. On the other hand, Arthur Riggs and colleagues thought of epigenetics as inheritable changes in gene expression that serve to regulate cell fate decisions and cellular phenotypes that cannot be explained by changes in DNA sequence [2]. While Waddington considered virtually all aspects of gene activity during development by which a phenotype arises, Riggs and colleagues refrained from defining any kind of mechanism involved. This situation has rapidly changed over the last decades due to major conceptual advancements in understanding the role of epigenetic mechanisms. DNA methylation and posttranslational modifications of core histones serve to lay down epigenetic states at the level of the genetic blueprint (*i.e.*, the DNA) and will be briefly touched on in this review, whereas nucleosome positioning, and non-coding RNAs (ncRNA) among others [3] are proposed to facilitate the formation of epigenetic states.

The X-linked gene *MECP2* (methyl-CpG-binding protein) recognizes epigenetic states (e.g., methylated DNA and chromatin conformation) and confers various transcriptional functions upon chromatin and DNA binding [4]. Additionally, different forms of *MECP2* mutations have been identified as the genetic cause of a severe neurodevelopmental disorder in humans, named Rett syndrome [5]. These findings show that accurate interpretation of epigenetic states by regulatory factors is an important determinant for mental health and underscore the role of DNA methylation in inheritable disorders [6].

On the other hand, an increasing number of studies has provided strong evidence over the last decade that environmental conditions [7], including social experiences [8,9], can couple to enduring epigenetic effects on phenotype. Accordingly, epigenetic states are plastic and serve to mediate between a dynamically changing environment and the static genetic blueprint. Stimulus-driven epigenetic states can underpin a form of “molecular plasticity” that is thought to facilitate an organism’s capability to mount an adaptive response as part of multilayered gene-environment interactions [10].

Here, we will discuss recent findings showing that Mecp2 can also act to translate various environmental experiences into lasting epigenetic changes at genes whose products associate with distinct phenotypes in mice. Overall, we propose for MECP2 a more dynamic role by linking experience-related diseases to epigenetic bookmarking and ultimately, mental disease in human.

## 2. The Make-Up of Epigenetic Marks

Within the scope of this review, we will briefly discuss two main epigenetic regulations, modifications of DNA and DNA-bound histones, and refer the interested readers for an in depth review of these and additional mechanisms to a series of recent publications [3,11,12,13,14].

### 2.1. DNA Modifications

DNA methylation is a biochemical process whereby a methyl group (CH_3_) is stably and covalently bound to the nucleotide cytosine in the DNA. Until recently, this modification was thought to occur predominantly at CpG dinucleotides. Recent genome-wide bisulfite sequencing showed, however, accumulation of high amounts of non-CG methylation of the neuronal genome during development from fetal to young adult in rodents and humans [15]. In contrast to classical CpG methylation (see below) the function of non-CG methylation remains poorly understood.

A family of enzymes known as DNA methyltransferases (DNMTs), comprising DNMT1, DNMT3A, DNMT3B, and DNMT3L [16], catalyzes the transfer of methyl groups to the DNA. DNMT1 is recruited to replication foci during S phase, where it primarily methylates hemimethylated DNA in order to maintain the original methylation state. In contrast, DNMT3A and DNMT3B fulfill a role as *de novo* methyltransferases by recognizing unmethylated CpG dinucleotides as substrates. This function is supported by assembly with the homolog DNMT3L that lacks on its own catalytic activity [17].

CpG sites are in general depleted in the genome, except from punctuated stretches of DNA termed CpG islands (CGIs), where CpG content is high. Nearly 70% of all annotated promoters associate with a CGI and are in general methylation-free [18]. In contrast, CpGs outside CGIs are mostly methylated [19].

CGI methylation has been correlated with gene repression in a variety of ways (as discussed for MECP2 in more detail below)—an observation that inspired the commonly held view that DNA methylation operates primarily as a repressive epigenetic mark [20,21]. Recent genome-wide promoter analysis indicates, however, that most inactive promoters remain unmethylated arguing against the role of DNA methylation as an all-purpose mechanism in gene expression [22]. Moreover, high amounts of DNA methylation have been detected in intra- or intergenic regions and were associated with increased transcription and/or regulation of alternative promoter usage [15,22,23,24,25,26,27]. Together, these findings suggest that the relationship between CpG methylation and gene expression depends on genomic and cellular context.

**Figure 1:**
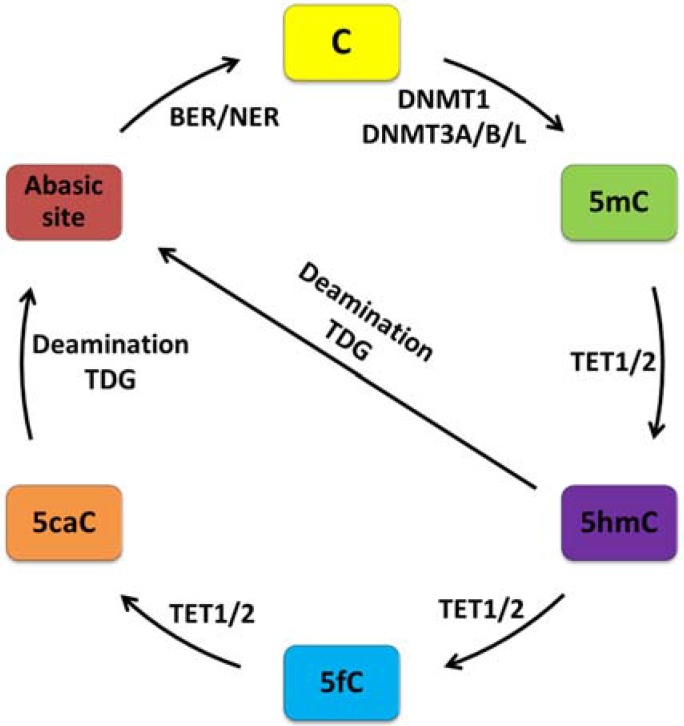
The life-cycle of DNA methylation in mammalian cells. The nucleotide cytosine (C) is methylated at the 5th carbon either by the DNA maintenance methyltransferase 1 (DNMT1) during DNA replication or *de novo* by DNA methyltransferase DNMT3A or DNMT3B. The catalytic inactive member DNMT3L enhances the activity of *de novo* DNMTs due to its scaffolding function. Active demethylation of 5-methylcytosine (5mC) can occur through iterative oxidation by ten-eleven translocation proteins (TET1/2) producing 5-hydroxymethylcytosine (5hmC), then 5-formylcytosine (5fC), and lastly 5-carboxylcytosine (5caC). These cytosine analogs may impair binding and activity of the maintenance methylation machinery in mitotically active cells and lead to passive dilution of oxidized 5mC derivatives. Alternatively, 5caC, but also 5hmC, can be efficiently deaminated to thymine and excised by thymine DNA glycosylase (TDG). Finally, the mismatched bases are repaired by the base excision and/or nucleotide excision repair machinery (BER/NER).

Our traditional view of DNA methylation as a relative stable, covalent modification introduced during development has to be revised following the recent discovery of active demethylation [28]. Initially, a number of oxidation-independent mechanisms were thought to control active demethylation (*i.e.*, nucleotide excision (NER) repair to erase 5-methylcytosine (5mC), direct base excision repair (BER) of 5mC by DNA glycosylases, and deamination and repair of 5mC among others). Yet, these biochemical reactions are unlikely to operate under physiological conditions as discussed elsewhere [28]. Instead, the transformative discovery of the ten-eleven (Tet) translocation family of proteins showed that this group of enzymes can catalyze the oxidation of 5mC into 5-hydroxymethylcytosine (5hmC) [29,30], and into further oxidized derivatives (5-formylcytosine (5fC) followed by 5-carboxylcytosine (5caC)) [31,32]. These oxidation products seem to impair binding and/or activity of the maintenance methylation complex and enhance replication-dependent passive dilution of oxidized 5mC (Figure 1). This process has been suggested to occur in mitotically active tissues, such as demethylation of the paternal genome during preimplantation development and in developing primordial germ cells (PGCs).

On the other hand, AID/APOBEC proteins may directly deaminate 5hmC to generate 5-hydroxyuracil (5hmU) and lead to the repair of the resulting 5hmU∙G mismatch by the DNA glycosylases TDG (thymine DNA glycosylase) and SMUG1 (single-strand selective monofunctional uracil DNA glycosylase) [33]—a pathway proposed to exist in mouse brain [34] (Figure 1).

Moreover, oxidized cytosines can also be directly erased by the DNA repair machinery. The derivatives 5fC and 5caC are efficiently excised by TDG and repaired by the base excision (BER) pathway; a possibility that has been described so far only in embryonic stem cells (ESC) [32,35].

Interestingly, oxidized derivatives of 5mC occur throughout the genome at specific regulatory sites [15] and may thus counterbalance the functions of 5mC by restructuring and/or disrupting interactions with associated reader and effector proteins [36].

Overall, these findings make it necessary to revise our perception of DNA methylation being stable. The methylome is dynamic in terms of genomic distribution of 5mC, cycles of cytosine methylation, iterative oxidation, replication-dependent dilution, DNA glycosylase-initiated base repair, genomic distribution of oxidized 5mC derivatives, and variable recruitment of readers and effectors.

### 2.2. Histone Modifications

In its native state DNA is wrapped around an octamer of histone proteins formed by two copies each of the core histones H2A, H2B, H3 and H4 or their variants [37]. This structure, the so-called nucleosome, is the building block of chromatin and becomes further condensed with the help of linker histone H1 and other non-histone proteins to produce condensed chromatin fibers largely inaccessible to the transcriptional machinery [38]. Nucleosomes are not only critical for tight packaging of DNA but also interfere with the assembly of transcription initiation complexes and progression of RNA polymerase II (PolII)-driven gene transcription [37].

Over the last years, various chromatin-remodeling enzymes have been identified, which can enable or disable transcription by disentanglement of nucleosomes in an ATP-dependent manner or by mobilizing the histone core relative to the DNA (*i.e*., nucleosome “sliding”) to expose regulatory DNA sequences [39]. Furthermore, the amino-terminal tails of the four core histones serve as substrates for a variety of enzyme-dependent, posttranslational modifications at selected amino acids, including lysine acetylation, lysine and arginine methylation, serine phosphorylation, and covalent binding of the small peptide ubiquitin among others [40]. Such modifications are of great interest, given available evidence that they endow nucleosomes with more functions than wrapping DNA by storing epigenetic information that regulates how genes are switched on and off and how expression patterns are maintained during cell division [41].

## 3. *MECP2* Mutations Encode a Heritable Neurodevelopmental Disorder

DNA methylation often couples to transcriptional repression by affecting gene expression through two routes: directly, by changing sequence-specific binding of transcription factors to their DNA address code (e.g., blocking or enhancing DNA binding of an activator or repressor, respectively) or indirectly, through recruitment of proteins, which recognize specifically methylated DNA to subsequently regulate gene expression. The search for latter proteins led to the discovery of MECP2, which became the founding member of a family of proteins comprising four additional members, called methyl-CpG-binding domain (MBD) proteins 1–4. Except MBD3, all of them share the conserved DNA binding domain through which they bind to methylated CpG residues [42].

### 3.1. MECP2 Mutations and Rett Syndrome

*MECP2* localizes to the X-chromosome at Xq28 and is in its mutated form the genetic cause of a postnatal, neurodevelopmental disorder named Rett syndrome (RTT, MIM 312750) [5]. More than 95% of the patients suffering from key features of RTT have mutations that mostly arise *de novo* in the paternal germline and include missense, nonsense, and frameshift mutations. Among these, 65% can be attributed to eight recurrent missense or nonsense mutations within the MBD strengthening its central role for MECP2’s function.

RTT is a progressive disease with an incidence of 1 in 10,000 and manifests predominantly during early childhood in girls who initially develop inconspicuously up to 6–18 months. This period is followed by developmental stagnation (microcephaly, growth retardation, weight loss, and muscle hypotonia) and further decline as the syndrome progresses (stereotypic movements, social withdrawal and unresponsiveness, loss of language, ataxia, seizures, autonomic perturbations among other symptoms) [5]. This characteristic course suggests that the manifestation of RTT syndrome coincides with the period during postnatal development when sensory-driven neuronal activity refines cortical circuitry, indicating that RTT may relate to synaptic function. In accord with this view, various developmental synaptic alterations have been found in *Mecp2* null mice comprising an altered balance between excitatory and inhibitory synaptic transmission in the somatosensory cortex [43], decreases in the number of hippocampal glutamatergic synapses [44], and an extended period of visual cortical plasticity [45].

### 3.2. MECP2 Expression and Neuropathological Changes

*MECP2* spans four exons, which encode two different protein isoforms differing in their N-termini as a result of alternative splicing of exon 2 [46]. The more abundant MECP2-e1 isoform (encoded by *MECP2α*) includes 24 amino acids encoded by exon 1 and lacks nine amino acids encoded by exon 2. Alternatively, the start site of the MECP2-e2 isoform (encoded by *MECP2β*) localizes to exon 2.

In general, MECP2 protein is less abundant in peripheral tissues than the brain where the longer transcript prevails and is highly expressed during embryonic development. In the rodent brain, Mecp2 expression is first found in the spinal cord and brainstem on embryonic day 12 (E12), followed by thalamus, hypothalamus, hippocampus, caudate putamen, and cerebellum (E14-16) [47,48]. The timing of MECP2 expression in mouse and human correlates with the maturation of the central nervous system, with ontogenetically older structures (*i.e.*, spinal cord and brainstem) preceding newer ones (*i.e*., hippocampus and cerebral cortex) indicating a role in the modulation of the activity or plasticity of mature neurons [49]. In support of this view, *Mecp2* null mice and RTT patients show an overall decrease in the size of the brain due to reduced neuron size, axonal and dendritic processes, cytoskeletal proteins and spine density among other changes that ultimately result in an impaired synaptic plasticity—a widely accepted cellular basis for learning and memory formation [5]. RTT brains do not display signs from degeneration, inflammation, gliosis or migration defects arguing against a neurodegenerative process and thus strengthen the concept of a disturbed postnatal neurodevelopment.

### 3.3. MECP2 DNA Binding and Transcriptional Regulation

MECP2’s MBD controls binding to symmetrically methylated CpG dinucleotides. Structural analysis of DNA-bound MECP2 complexes by X-ray crystallography revealed that the specificity for the cytosine methyl group derives from multiple contacts, whereby some of these are mediated by immobilized water molecules in the major groove of the double helix [50]. Moreover, MECP2 contains two consensus A/T-hook motifs, which can bind to the minor groove of AT-rich duplex DNA. This property has been suggested to underpin MECP2’s high-affinity DNA binding to methyl CpG residues in the context of A/T-rich sequence motifs [51].

Upon binding to methylated DNA, MECP2 recruits via its central transcription repression domain the co-repressors Sin3a and histone deacetylases (HDACs) 1 and 2 [52,53], which promote histone deacetylation and chromatin compaction. Additional repressor mechanisms include direct interactions between MECP2’s C-terminal domain and chromatin [54] and/or the recruitment of other factors known to modulate gene expression, such as the ATPase responsible for α-thalassemia/mental retardation syndrome X-linked (ATRX) [55], DNMT1 [56], histone methyltransferase Suv39H1 [57], and the corepressors c-Ski and N-CoR [58] among others. Moreover, MECP2 also interacts with the RNA-binding protein Y box-binding protein 1 (YB1), suggesting also a role in RNA splicing [59].

Chromatin immunoprecipitation experiments combined with next generation sequencing (ChIP-seq) revealed Mecp2 binding patterns tracking genome-wide CpG methylation density in mice [60].

Despite this clear evidence, MECP2’s preference for methylated DNA has been questioned by the finding that it can also associate with nucleosomes containing unmethylated DNA and compact local chromatin [61]. This observation is further supported by recent genome-wide ChIP-seq analysis of a human neuroblastoma cell line that revealed binding to unmethylated and methylated DNA [62]. Moreover, ChIP analysis of single loci showed that mouse Mecp2 can bind, possibly by partnering with pre-bound proteins, to the unmethylated promoter of *Slc6a2* or the unmethylated allele of imprinted *H19* [63,64].

Taken together, these findings raise the possibility of additional mechanisms apart from DNA methylation to govern MECP2’s DNA binding activity.

### 3.4. MECP2 Target Genes

Transcriptional profiling of brain tissues from *Mecp2* null mice revealed only minor changes in gene expression indicating that Mecp2 may not operate as the global repressor originally thought but rather as a local factor [65]. These results have been corroborated in subsequent studies of global gene expression changes in various models of *Mecp2* null mice, both for gene transcripts and micro RNAs as recently reviewed elsewhere [66,67] and prompted the suggestion that regulation of selected genes in specific neuronal populations may escape detection in case of bulk tissue analysis (e.g., glia cells express only low amounts of Mecp2). Consistent with this hypothesis, region-specific analyses of the hypothalamus and cerebellum [68,69] showed numerous subtle changes in gene expression. Even so, many of them were shared across tissues and therefore disfavored a role of Mecp2 in site-specific regulation of a confined gene set. Likewise, another study at different time periods in Mecp2 mice models felt short in identifying a set of consistently altered genes in the cerebellum [70].

Since Mecp2 is expected to bind *per se* methylated DNA, the presence of Mecp2 at deregulated genes as inferred from ChIP experiments does not implicate a causal role in their regulation. Therefore, expression changes before manifestation of disease symptoms may help to distinguish between primary and secondary effects; however, few changes have been reported so far [70,71,72]. The work of Nuber and co-workers, who studied expression changes in pre-symptomatic, early-symptomatic, and late-symptomatic *Mecp2* null mice, is of particular interest in the context of this review. The authors reported upregulation of several glucocorticoid-regulated genes including serum glucocorticoid-inducible kinase 1 (*Sgk1*), FK506-binding protein 5 (*Fkbp5*), and pro-opiomelanocortin (*Pomc*—the polypeptide precursor of ACTH). These genes play important roles in neuronal plasticity, fast intracellular feedback inhibition of glucocorticoid receptor signaling, and activation of the hypothalamic-pituitary-adrenal (HPA) axis in response to stress (see below) [73,74,75]. Expression changes in these genes were measured across the entire investigation period in *Mecp2* null mice and ChIP analysis in wild type mice corroborated Mecp2 binding to the respective regulatory regions in brain (*i.e.*, *Sgk1* and *Fkbp5*) [72] and anterior pituitary (*i.e.*, *Pomc*) as reported elsewhere [76]). Noteworthy, *Mecp2* null mice showed only slightly elevated serum glucocorticoid levels. Because *Sgk1* and *Fkbp5* are induced by glucocorticoids, while *Pomc* is inhibited, these results argue against a critical role of glucocorticoids in deregulation of these genes but support a local role of Mecp2.

Further support for Mecp2’s role in gene repression and the stress response has been gained by McGill *et al*. [77], who chose a phenotype-driven approach in mice to identify Mecp2 target genes relevant to RTT pathology. Mice harboring a truncated Mecp2 allele (Mecp2^308/Y^) showed enhanced anxiety-like behavior similar to common high-anxiety episodes in RTT patients and an increased stress response as evidenced by higher serum corticosterone levels. Behaviorally, the hypothalamic neuropeptide corticotrophin-releasing hormone (CRH), a major driver of the HPA axis, induces anxiety, while CRH receptor-1 antagonists or genetic ablation of the receptor reduce anxiety [78]. Consistent with the behavioral phenotype of Mecp2^308/Y^ mice, Crh mRNA expression was upregulated in the paraventricular nucleus of the hypothalamus (PVN), the central amygdala, and the bed nucleus of the stria terminalis; all of these regions are important for controlling the stress response and anxiety. Crh upregulation in Mecp2 deficient mice occurred exclusively in those neurons where it is normally expressed but not at ectopic sites, and suggests to Mecp2 a role as modulator rather than an on-off switch of gene expression. While there were no differences in CpG methylation between wild type and mutant mice at the Crh promoter, ChIP analysis revealed robust binding of wild type Mecp2, but not of its truncated version [77].

Although above ChIP experiments left little doubt that Mecp^308^ was poorly bound *in vivo* to the *Crh* promoter, a limitation of this work is that the truncated Mecp2 protein lacks key serine residues (S421 and S424) that can serve as substrate for neuronal activity-dependent phosphorylation and dissociation from methylated DNA [79]. In this respect, a further report seems to cast doubt on Mecp2’s role as repressor in its wild type state [80].

Clinical studies have shown that autism and comorbid anxiety are common in boys carrying *MECP2* duplications and indicate that either decreased or increased amounts of MECP2 are critical to neuronal functions [81]. In support of this hypothesis, doubling Mecp2 levels in mice causes heightened anxiety, autism-like features, and upregulation of *Crh* and *Oprm1* (a opioid receptor subtype) in mice [80]. Reductions in the expression of Crh or its receptor, Crhr1, attenuated anxiety-like behavior, whereas reductions in the expression of Oprm1 improved social behavior. Together, these data assign to Mecp2 a role in the regulation of molecular pathways important to anxiety and social behavior and raise the question whether Mecp2 possibly activates Crh expression.

In view of these discrepancies, it is important to note that both mice models did not restrict Mecp2 misexpression to the PVN and therefore cannot dissect system-wide effects on neuronal activity from local ones in terms of Crh transcription. This situation is reminiscent of Mecp2’s controversial role in the regulation of brain derived neurotrophic factor (*Bdnf*), where both repressive and activating effects have been reported to occur in a context-dependent manner as reviewed elsewhere [82]. The advent of temporospatial specific knock-out models may help to clarify and reconcile such disparate findings. For the present, we note that Mecp2 and neuronal function influence each other in their activities; an important insight, which does not interfere with our intention to discuss Mecp2’s potential role in epigenetic programming of intact physiological systems.

## 4. Neuronal Activity Controls Mecp2 via Posttranslational Modifications

Next, we will discuss growing evidence for a role of neuronal activity in regulating Mecp2’s functions in mice. Neuronal activity-dependent changes in structure and circuitry underlie neuronal plasticity, an essential feature of the brain, coordinating various processes like learning and memory and the ability to cope with and adjust to dynamically changing environments. Recent evidence also suggests neuronal activity-driven changes in DNA modifications, in particular CpG methylation, to constitute a new layer of molecular plasticity whereby both cellular and molecular plasticity are likely to influence each other in deposition and maintenance [10].

Until now, Mecp2’s transcriptional role in cellular and molecular plasticity remains poorly defined, although, an increasing number of posttranslational modifications have been identified (as reviewed elsewhere [4,83,84]). Among these, protein (de-) phosphorylation has been most thoroughly studied and is of particular interest to Mecp2’s role in epigenetic programming (see below).

### 4.1. Activity-Dependent (De-)Phosphorylation of Mecp2

The concept of Mecp2 phosphorylation dates back to two key studies from 2003, which firstly showed that neuronal activation triggers calcium (Ca^2+^)-dependent phosphorylation of Mecp2 in mice and rat, dissociation from the *Bdnf* promoter III, and consequently enhanced transcription [85,86]. These findings stimulated the hypothesis that MECP2 participates in neuronal activity-driven gene expression and may thereby contribute to RTT pathology. The responsible amino acid residue was mapped to S421 and is phosphorylated on average at 10%–30% of all Mecp2 molecules [79].

Another interesting phosphorylation site of Mecp2 refers to S80 [87] (note; designation of amino acids refers to mouse Mecp2-e2). In contrast to S421 phosphorylation, S80 is dephosphorylated as a result of neuronal activity-dependent Ca^2+^ influx. Although both phosphorylation sites are subject to Ca^2+^-dependent signaling, either modification can occur on its own. Furthermore, they mediate opposite effects on Mecp2 binding: phosphorylation of S80 enhances Mecp2’s association with chromatin [87], while phosphorylation of S421 promotes dissociation from target genes [79].

Replacement of S80 by alanine (S80A), thus abolishing phosphorylation, weakened Mecp2’s presence at a number of candidate target genes, including *Pomc*, in neural cultures treated with tetrodotoxin (an inhibitor of neuronal activity) but did not affect gene expression. This finding indicates that S80 phosphorylation may need to operate jointly with other posttranslational modifications to regulate transcription more robustly. Compatible with this suggestion, *Mecp2^S80A^* knock-in mice displayed a distinct phenotype comprising reduced locomoter activities reminiscent of RTT patients [87].

The homeodomain-interacting protein kinase 2 (HIPK2) catalyzes S80 phosphorylation [88]. Forced expression of both proteins induces apoptosis in wild type and *Mecp2* deficient mouse embryonic fibroblasts and prompts the question whether Mecp2 has a related function during neurodevelopment. Lastly, S80 phosphorylation has been reported to enhance the RNA-dependent interaction with YB-1 through which Mecp2 regulates RNA splicing [89].

Expression of phosphorylation-defective Mecp2 S412A in primary rat neurons blocked the effect of wild type Mecp2 on dendritic patterning and dendritic spine morphogenesis, two activity-mediated processes that have been linked to RTT on the basis of neuropathological studies of RTT patients and *Mecp2* null mutant mice. Moreover, phosphorylation-defective S421A knock-in mice (Mecp2^S421A/y^) revealed defective patterning of distal apical dendrites pointing to a role of Mecp2-S421 phosphorylation in circuit development [79].

### 4.2. Mecp2 Phosphorylation in Learning and Memory

Abrogation of S421-phosphorylation in Mecp2^S421A^ mice causes enhanced dendritic complexity of cortical neurons and a shift in excitation-inhibition balance towards cortical inhibition reminiscent of *Mecp2* null mice [43,90]. Moreover, behavioral responses to new versus familiar mice are reduced in Mecp2^S421A^ mice. Even so, interest in new mice is preserved in Mecp2^S421A^ mice pointing to a deficit in processing new experiences rather than in social recognition. On the other hand, anxiety-like behavior, spatial learning, and memory appeared largely unaffected in Mecp2^S421A^ mice [90].

A related study at the same time reported a strong increase in Mecp2-S421 phosphorylation following hippocampus-dependent learning tests in mice [91]. Since previous mass spectrometric analysis of seizure-induced Mecp2 phosphorylation had detected two neighbored, possibly co-regulated phosphorylation sites, namely S421 and S424, [87], a knock-in mouse carrying serine-to-alanine substitutions at both sites (Mecp2^S421A/S424A^) was created [91]. Unexpectedly, these mice showed an increased learning ability, which associated with enhanced NMDA receptor-dependent Schaffer collateral-CA1 and mossy fiber-CA3 long-term potentiation (LTP) in hippocampal slices and an increased number of excitatory synapses in cultured hippocampal and cortical neurons.

Taken together, these studies jointly point to the importance of Mecp2 phosphorylation for learning processes, although the relevance of single versus multiple phosphorylation sites remains still unresolved and may differ in a cell type and tissue-specific manner.

With respect to transcriptional regulation, Mecp2^S421A/S424A^ occupancy was increased at several target genes and conferred either repression (glutamate receptor 1 (*Grm1*), myocyte enhancer factor 2c (*Mef2c*)) or transactivation (*Bdnf*, bone morphogenetic protein 4 (*Bmp4*)). The latter result is in accord with two related studies [68,90], indicating that DNA-bound Mecp2 can *per se* activate target genes.

Overall, differences in learning and memory between single and double phosphorylation-defective *Mecp2* mutations, *Mecp2* null, and overexpressing mice are incompletely understood [83]. Temporospatial differences in Mecp2 expression and the response to neuronal activity between wild type and various transgenic mice are likely to determine phenotypic outcomes across a lifetime and necessitate further investigations.

### 4.3. Mecp2 Phosphorylation in Drug Addiction

Drug abuse leads to long-lasting changes in behavior by altering the expression of genes underlying the function and plasticity of reward-associated brain circuits at the cellular and synaptic level [92]. A major reward system, the mesolimbocortical dopamine circuit, consists of dopaminergic neurons in the ventral tegmental area (VTA) and their synaptic connections in the nucleus accumbens (NAc), frontal cortex, and associated limbic structures [93].

Repeated exposure to psychostimulants (e.g., amphetamines, cocaine) elicits changes of striatal synapses (*i.e*., in the caudate putamen and NAc), thought to regulate behavioral sensitization and conditioned place preference (CPP). In this respect, repeated administration of cocaine has been shown to increase Mecp2 protein expression in a subset of neurons in the dorsal caudate-putamen, dentate gyrus, and frontal cortex [94]. Moreover, acute viral-mediated Mecp2 knock-down in the adult NAc increased amphetamine-induced locomotion and CPP, whereas Mecp2 overexpression inhibited CPP [95]. Together, these results suggest that Mecp2 confines the rewarding effect of psychostimulants, whereby local alterations in Mecp2 expression within the mesolimbocortical circuit may regulate associated behavioral responses.

Notably, a single systemic application of cocaine triggered a transient increase in Mecp2-S421 phosphorylation in the striatum, but not in the medial prefrontal cortex [96]. Cocaine-stimulated Mecp2 phosphorylation in the caudate putamen, but not in the NAc, was significantly attenuated by pretreatment with an N-methyl-d-aspartate (NMDA) glutamate receptor antagonist linking this receptor type to site-specific Mecp2-S421 phosphorylation.

Similarly, amphetamine treatment caused a fast, dopamine-dependent phosphorylation of Mecp2-S421 [97]. This event occurred selectively in D1-class dopamine receptors containing GABAergic interneurons within the NAc pointing to a mechanism whereby psychostimulants may control Mecp2 function.

Psychostimulants, like cocaine or amphetamine, act as indirect monoamine receptor agonists by inhibiting and/or reversing the function of monoamine transporters and consequently rapidly increase extracellular levels of dopamine, serotonin, and norepinephrine. Among these neurotransmitters, increased levels of norepinephrine felt short to induce Mecp2-S421 phosphorylation, whereas dopamine and serotonin independently of each other lead to S421 phosphorylation in selective brain regions [98]. Moreover, a combination of pharmacological agents acting at specific dopamine and serotonin receptor subtypes reproduced amphetamine-dependent Mecp2-S421 phosphorylation patterns in a brain region and cell-type specific fashion.

In further support of these findings, Mecp2^S421A^ knock-in mice have been recently reported to show a reduced threshold for locomotor sensitization following amphetamine application and an increased behavioral response to the reinforcing effects of self-administered cocaine [97]. This behavioral phenotype associated at the cellular level with changes in medium spiny neurons intrinsic excitability and immediate early gene expression (*i.e.*, *Creb* and *fos*), characteristic of repeated intake of these drugs. Collectively, these findings strengthen the hypothesis that Mecp2-S421 phosphorylation serves to restrain circuit plasticity in the NAc and consequently the development of addictive-like behaviors.

Overall, these results show tissue- and cell-type specific Mecp2-S421 phosphorylation in response to acute and repeated psychostimulant intake, whereby Mecp2 expression levels have a critical role in balancing behavioral outcomes. Mecp2-S421 phohorylation is mediated by specific intracellular signaling pathways following activation of the respective neurotransmitter receptor systems.

A major strength of this set of studies is the emphasis on wild type mice together with site-specific acute manipulations of Mecp2 expression levels; an approach that overcomes many of the concerns from transgenic mice discussed above. A cutback is, however, the fact that despite strong evidence for a role of Mecp2 in coupling drug experience to behavior, the molecular underpinnings of psychostimulant-induced plasticity changes in reward-associated brain circuits remain unknown. Since above data also suggest that neural circuit-stimulated Mecp2-S421 phosphorylation may constitute a mechanism to confer specificity on its transcriptional activities *in vivo*, future studies should assess the effects of Mecp2-S421 phosphorylation on target genes in drug addiction at the circuit level using candidate or genome-wide analysis.

### 4.4. Mecp2 Phosphorylation in Mood Disorders

Tricyclic antidepressant and selective-serotonin reuptake inhibitors (SSRI) were originally thought to operate in major part through activation of monoamine receptors to alleviate symptoms of major depressive disorders (MDD) [99]. Improvements in depressive symptoms take, however, several weeks and require continuous drug administration irrespective of their rapid effects on increasing extracellular levels of monoamine neurotransmitters (e.g., serotonin (5-HT) and norepinephrine). These findings have stimulated the hypothesis that antidepressants additionally elicit long-lasting changes in neuronal gene expression important to synaptic plasticity within circuits that underpin major depression [99]. As discussed before, psychostimulants activating dopamine or 5-HT receptors trigger Mecp2-S421 phosphorylation in specific populations of neurons in the NAc. Interestingly, this finding can be reproduced by administration of the SSRI citalopram, but not by reboxetine, a selective noradrenaline reuptake inhibitor [97,98]. Together, these findings raise the possibility that MECP2 phosphorylation in humans may be involved in both 5-HT-dependent depressive-like symptoms as well as their alleviation under antidepressant therapy. In support of this view, application of the tricyclic antidepressant imipramine, an inhibitor of serotonin and norepinephrine transporters, selectively stimulated Mecp2-S421 phosphorylation in the NAc and the lateral habenula [98], two brain regions important for depressive-like behavior in mice [100]. While previous behavioral profiling of wild type and Mecp2^S421A^ mice revealed no differences in motor function, social interaction, or anxiety-like behaviors [90], additional analyses in the tail-suspension and forced swim test evidenced an enhanced sensitivity to environmental stressors in S421-phosphorylation deficient mice. Acute administration of imipramine reduced immobility in both groups in the forced swim test indicating that loss of Mecp2 phosphorylation did not preclude an immediate pharmacological response. In contrast, following chronic social defeat stress chronic administration of imipramine largely restored social interaction in wild type, but not in Mecp2^S421A^ mice.

Overall, these findings reveal that molecular pathways responsible for acute responses to imipramine are preserved in Mecp2^S421A^ mice, but seem to be disrupted in case of long-term responses. Therefore, Mecp2-S421 phosphorylation may participate in imipramine-induced transcriptional effects that regulate neuronal plasticity in those circuitries that are targeted under chronic imipramine administration.

A puzzling result from these studies is the fact that Mecp2-S421 phosphorylation can counteract the effects from both reward and stress by acting in the same neuronal circuitry of the NAc. An explanation of this finding is the possibility that both reward and depression-related behaviors depend on the specific circuit context under which they are activated. Alternatively, since Mecp2^S421A^ mice lack S421-phosphorylation throughout the whole brain from development to adulthood, the missing response to chronic imipramine treatment may involve other, still unidentified, circuitries interacting with those from the NAc.

## 5. Mecp2 Mediates Early-Life Stress

### 5.1. Early-Life Stress

Early life adversity has been defined by the National Comorbidity Survey Replication Study [101] as a variety of conditions, including interpersonal loss (parental divorce or death and other separation from parents or caregivers), parental maladjustment (criminality, violence, substance abuse, and mental illness), maltreatment (physical or sexual abuse and neglect), life-threatening childhood physical illness in the respondent, and extreme childhood family economic discrimination. The National Center of Child Abuse and Neglect reported approximately 3.4 referrals across the US in 2009 [102], which corresponds to a victim rate of 9.1 victims per 1000 children in the US population. Among these, 78.5% suffered neglect, 17.6% physical abuse, and 9.1% sexual abuse.

Worryingly though, neglect during early childhood receives less publicity than abuse although it continues to be the most common form of maltreatment [103].

During early life, mother and infants engage in reciprocal dynamic interactions addressing the infant’s needs and the mother’s capacity to provide them. These relationships play a major role in shaping the child’s psychological, cognitive, and affective development, and its adaptive responses to stressful events [104,105]. Infants lacking maternal emotions are retarded in reaching developmental milestones, make less eye contact, smile less, are irritable, and become increasingly withdrawn, less responsive and less engaged, symptoms that reflect not only an aversive environment but also the absence of positive master experiences [106].

Epidemiological studies performed at the Center for Disease Control evidenced a strong dose-response relationship between childhood adversities and mental health in adulthood [107]. Accordingly, multiple adverse exposures during early life increase the risk for major depression by fourfold [108], whereby the severity of childhood adversity corresponds with lifetime recurrent depression [109] and suicidality [110]. Early life adversity also increases the risk for posttraumatic stress disorder (PTSD) in adults, to undergo re-exposure to trauma in adulthood, and to develop PTSD in response to adult trauma [111]. Consequently, early life adversity is a strong risk factor for major depression and PTSD, a protracted course, and a weaker response to therapy.

### 5.2. The HPA Axis Mediates Early-Life Stress

Animal and human data suggest that stress has the strongest influence on those structures that are developing at the time of stress exposure (*i.e*., in childhood) and those that are subject to age-related changes (*i.e.*, in adult and aged individuals) [112]. Stress in the perinatal period affects the development of many brain regions regulating the HPA axis like the hippocampus, the frontal cortex, and the amygdala, possibly leading to a protracted glucocorticoid response to stress that persists into adulthood. During adolescence, the frontal cortex undergoes major refinements and may be most vulnerable to the effects of stress during this period, whereas in adulthood and old age those brain regions that undergo the most rapid decline as a result of aging appear preferentially vulnerable to the effects of stress hormones.

Children deprived of nurture during early life develop structural and functional abnormalities in brain regions controlling cognition (e.g., prefrontal cortex, hippocampus), mood (anterior cingulate cortex), emotions (amygdala), and stress coping (hippocampus, hypothalamus) [113]. Stress activates the HPA axis, resulting in glucocorticoid secretion that must be set back to resting conditions to prevent the harmful effects of sustained levels on depression and anxiety [114]. ELS typically manifests with glucocorticoid (GC) hypersecretion that may mediate an enhanced risk for depression and anxiety [115,116]. Each episode of depression increases the risk and severity of subsequent episodes, indicating that the initial event that triggered the onset of depression leaves a dormant mark of vulnerability that is remembered and executed or reinforced with subsequent relapses contributing to the progressive course of the disease over life time [75].

**Figure 2:**
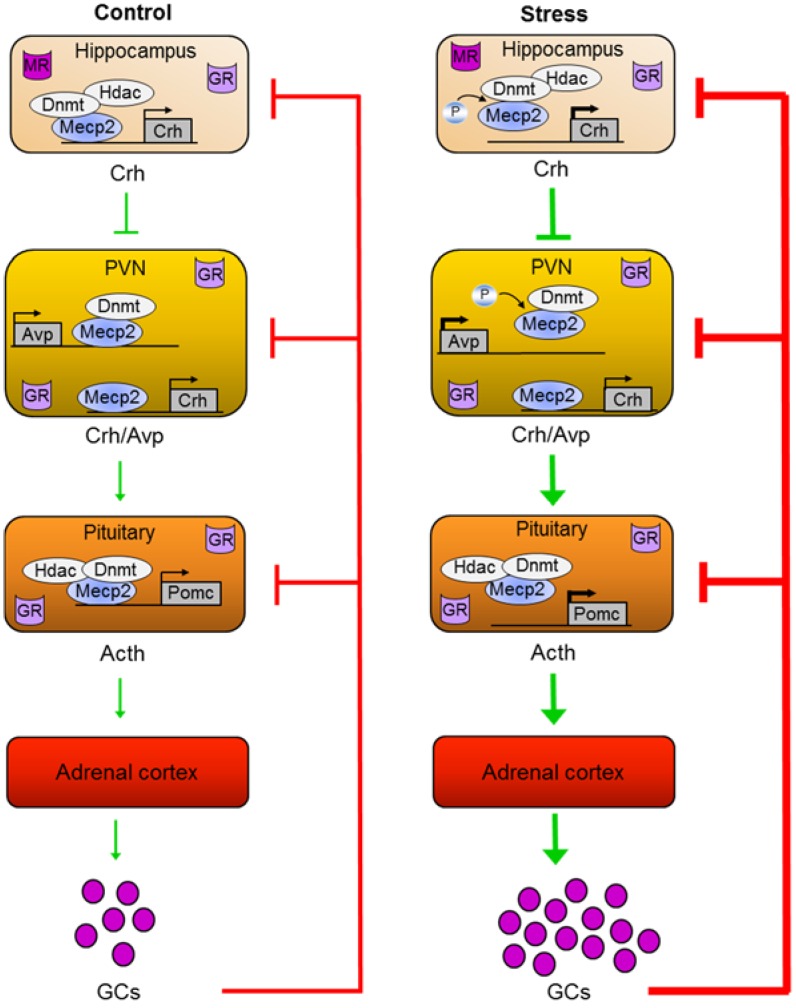
Roles of Mecp2 in epigenetic programming of the HPA axis by ELS. The two neuropeptides corticotrophin-releasing hormone (Crh) and arginine-vasopressin (Avp) are released in response to stress from the nucleus paraventricularis of the hypothalamus (PVN) and jointly stimulate in the anterior pituitary the production of pro-opiomelanocorticotrophin precursor mRNA (Pomc) and the secretion of its posttranslational product adrenocorticotrophin (Acth). Subsequently, Acth enhances the secretion of glucocorticoids (GCs) from the adrenals. These stress hormones bind to nuclear glucocorticoid and mineralocorticoid receptors (GR and MR), which are expressed at different levels of the HPA axis and serve to set back the stress response. In hippocampal CA3 Mecp2 binds under resting conditions to the *Crh* promoter and represses gene activity through recruitment of Dnmts and Hdacs. Upon ELS exposure, Mecp2 becomes phosphorylated at S421, dissociates, and relieves *Crh* repression. Mecp2 also binds to *Crh* in the PVN but stays unaffected by ELS. In contrast, Mecp2 occupancy at the downstream *Avp* enhancer responds to ELS by S421-phosphorylation and derepression of *Avp*. Due to its association with Dnmts, Mecp2 occupancy maintains enhancer methylation, while ELS-induced dissociation facilitates hypomethylation and gives rise to a lasting memory trace. Likewise, Mecp2 binds to the proximal *Pomc* promoter region in the anterior pituitary and interacts hereby with Dnmts and Hdacs to repress *Pomc* expression. In contradistinction to the PVN, ELS-induced Mecp2 dissociation is unrelated to S421-phoshorylation indicating tissue- and cell-type specificity of this modification. Loss of Mecp2 binding facilitates, however, *Pomc* hypomethylation and increased gene expression.

A broad range of physiological and psychological stressors leads to activation of the HPA axis, culminating in the production of glucocorticoids by the adrenals [117]. Different brain regions, which perceive and process the respective stressors, innervate the paraventricular hypothalamus (PVN) and trigger the release of two hypothalamic neuropeptides, CRH and arginine vasopressin (AVP) (Figure 2). These neuropeptides are transported to the anterior pituitary via the hypopyhseal portal system to bind there to their respective G-protein coupled receptors. This event induces the synthesis of the precursor pro-opiomelanocorticotrophin (POMC) and secretion of its post-translational product adrenocorticotrophin (ACTH), which stimulates the adrenal cortex to secrete cortisol (in humans) and corticosterone (in humans, rat, and mice). Corticosteroids are lipophilic molecules that can easily passage the blood-brain barrier and bind to intracellular glucocorticoid (GR) and mineralocorticoid (MR) receptors (Figure 2). These act as ligand-gated transcriptional regulators and are coexpressed in neurons of the limbic system. High levels of GR additionally occur in the PVN and anterior pituitary, both of which are important sites for negative feedback regulation in addition to the hippocampus. Given its higher affinity for GCs, the MR is proposed to assess and initiate the onset of the stress response, whereas GR, requiring higher concentrations of GCs for activation, serve to limit and finally terminate the stress response.

### 5.3. A Role for Mecp2 in ELS-Dependent Epigenetic Programming of Avp

Periodic infant-mother separation (3 h per day from postnatal day (PND) 1–10) is an established model for ELS in mice [118]. We previously showed that ELS leads to enduring hyperactivity of the HPA axis, characterized by corticosterone hypersecretion under resting conditions and hyperresponsiveness to an acute stressor later in life. This neuroendocrine phenotype associated with distinct behavioral changes comprising memory deficits in an inhibitory avoidance task and increased immobility in the forced swim test [118].

While CRH plays an increasingly important role for the HPA axis as the organism matures, AVP is crucial for an appropriate adrenocortical response to stress during fetal development and perinatal life when the endocrine response to stress is dampened [119,120,121]. Consistent with this view, ELS resulted in an acute and long-lasting upregulation of *Avp*, but not of *Crh*, in the hypothalamic PVN. In contrast, Avp expression in the nucleus supraopticus, which mainly controls fluid homeostasis, remained unaffected by ELS [118].

Notably, *Avp* upregulation was accompanied by reduced DNA methylation at multiple CpG residues throughout the downstream enhancer region that localizes within a CpG island of intermediate CpG density and plays an important role in tissue-specific expression. ELS-induced hypomethylation was strongest at six weeks and three months and declined one year after the initial event.

Interestingly, CpG residues most responsive to ELS matched the criteria of high affinity MECP2 DNA binding sites [51] indicating a role for Mecp2 in experience-dependent epigenetic programming of *Avp*. A ChIP scan of the entire *Avp* locus with antibodies against different MBD proteins revealed that Mecp2 was selectively enriched at the enhancer region in naïve mice [118]. In accord with a reduced enhancer methylation, Mecp2 binding was diminished in young adult ELS treated mice when compared to controls. Unexpectedly, similar results were obtained at PND 10 following termination of ELS suggesting that signals unrelated to DNA methylation control Mecp2 binding to the enhancer region. This finding raised the possibility that ELS-dependent neuronal activation may trigger Mecp2 phosphorylation, dissociation from the enhancer, and derepression of *Avp*. In agreement with this hypothesis, we detected increased Mecp2-S421 phosphorylation and calmodulin kinase II phospho-immunoreactivity in Avp positive neurons at PND 10 in ELS mice. The phosphorylated state corresponds to the active form of this kinase and has been suggested to couple neuronal activity-driven Ca^2+^ influx to Mecp2-S421 phosphorylation [79,87]. Mecp2-S421 phospho-immunoreactivity declined after termination of maternal separation and was largely undistinguishable in adult mice. This result suggests that Mecp2 dissociation from the *Avp* enhancer is initially under the control of neuronal activity-dependent phosphorylation until DNA hypomethylation becomes the leading force.

Two recent studies postulated for DNA-bound Mecp2 a role in gene activation both at the local and genome-wide scale in the hypothalamus [68,80]. This raises the question whether Mecp2 binding actually confers repression to *Avp* in the PVN. Sequential ChIP experiments with antibodies against active or repressive histone 3 marks followed by immunoprecipitation with an antibody against Mecp2, showed that Mecp2 preferentially associated with repressive histone marks [122]. In support of this result, Mecp2 binding at the *Avp* enhancer was accompanied by recruitment of *de novo* Dnmts (Dnmt3a and Dnmt3b), but not Hdacs (Hdac1 or Hdac2). Such selectivity is in agreement with a previous report [56] and may also depend on tissue type (see below).

Extensive methylome reconfiguration takes places during development from fetal to young adult and whole-genome detection of 5-hmC revealed that this mark is present in fetal brain cells at locations that loose CG methylation and become activated during development [15]. In this respect, Mecp2 binding, Dnmt recruitment, and *Avp* enhancer methylation seem to calibrate postnatal Avp expression rather than to switch it off [122].

Overall, these experiments show that ELS in mice leads to the formation of a molecular memory at the *Avp* enhancer and point to a central role of Mecp2 in the mediation of experience-dependent epigenetic programming. ELS-induced depolarization of parvocellular neurons leads to Mecp2 phosphorylation, detachment from the enhancer, and derepression of *Avp*. Since Mecp2 serves as a platform for the recruitment of Dnmts, loss of enhancer binding facilitates DNA demethylation during postnatal methylome reconfiguration. Consequently, lasting enhancer hypomethylation underlies reduced Mecp2 binding and leaves an enduring memory trace of the initial stimulus (Figure 2).

### 5.4. A Role for Mecp2 in ELS-Dependent Epigenetic Programming of Crh

A number of recent studies reported that prenatal, postnatal, or adult stress associates with hypomethylation at the proximal *Crh* promoter in the rodent PVN and central amygdala as reviewed elsewhere [111]. The outcome on mRNA levels was, however, more inconsistent and both methylation and expression changes depended partly on the sex of the animals [123]. In our own work, ELS did not affect Crh expression in the male PVN or central amygdala [118], although, Mecp2 occupied the proximal promoter in association with repressive histone marks as reported previously [77]. This result can be explained by the finding that Mecp2 phosphorylation depends on the activation of specific neural circuits differing between Avp or Crh expressing neurons and/or different genomic context of *Avp* enhancer and *Crh* promoter regions in Avp and Crh coexpressing neurons. Therefore, these results are consistent with the specificity of Mecp2 phosphorylation both at the circuit and gene level.

In the developing hippocampus Crh is produced in several cell populations including Cajal-Retzius cells, while a more restricted pattern is observed in adult rodents, where most of the peptide is produced and stored within interneurons residing in the pyramidal cell layers of the CA1 and CA3 areas and is released from axon terminals during stress [124]. Crh has time- and dose-dependent effects on learning and memory via modulation of synaptic function and plasticity. Short, physiological increases in Crh act within the range of seconds to minutes to facilitate memory formation. In contrast, protracted, strong elevations of this neuropeptide cause spine retraction and loss of synapses impairing learning processes and ultimately, premature cognitive decline [125,126].

Maternal separation in rat led to impaired hippocampal glutamatergic synaptic plasticity and memory deficits in adults. Both effects were efficiently blocked by a Crh receptor 1 antagonist and correlated with increased Crh mRNA and protein expression in hippocampal CA1 [127]. Interestingly, Mecp2 occupancy was significantly reduced at the *Crh* promoter in the hippocampal CA1 region of maternal separated rats concomitant with increased Mecp2-S421 phospho-immunoreactivity (Figure 2). In support of a repressor function, sequential ChIP with antibodies against Hdac2 or Dnmt1 evidenced an interaction with Mecp2 [127]. Moreover, environmental enrichment ameliorated the long-term behavioral effects of ELS, prevented upregulation of hippocampal Crh and Mecp2-S421 phosphorylation, and reinstated Mecp2 binding to the *Crh* promoter in maternal separated rats [127].

Overall, these findings support a role of Mecp2 in mediating the effects of ELS on hippocampal memory deficits via epigenetic programming of *Crh* and strengthen the tissue-, cell-type, and circuit specificity of Mecp2-S421 phosphorylation.

### 5.5. A Role of Mecp2 in ELS-Dependent Epigenetic Programming of Pomc

AVP and CRH are produced in neurons of the PVN, which sent their axons to the median eminence. The primary capillary plexus in the upper infundibulum receives these neuropeptides from the axon terminals and carries them via the portal veins to the adenohypophysis. Binding to their respective membrane-bound receptors triggers an increase in the intracellular levels of cAMP and Ca^2+^. Together, these second messengers drive expression of pituitary *POMC* and stimulate secretion of its posttranslational product ACTH.

Consistent with an increased Avp secretion, we detected higher levels of Pomc mRNA under resting conditions in ELS mice and also measured an enhanced secretion of Acth following application of the Avp/Crh challenge test [76].

At the molecular level, these findings were associated with hypomethylation of multiple CpG residues in the proximal *Pomc* promoter region, which persisted for at least one year. Among the CpG residues modulated by ELS, those that were previously shown to be critical for both *Pomc* gene expression and activation by upstream secretagogues, such as Crh and Avp, showed a particularly strong and sustained alteration in methylation levels (*i.e*., CpG 6-16). Transfection assays of patch-methylated *Pomc* promoter constructs further supported the functional role of this region in gene expression and conferred efficient repression by Mecp2, but not by other MBDs [76]. Additional electrophoretic mobility shift assays and mutational experiments showed that the ELS-responsive region encoded several high-affinity Mecp2 DNA binding sites [51]. Consistent with these findings, *in vivo* ChIP experiments showed that Mecp2 occupied the proximal *Pomc* promoter region in the anterior pituitary and associated hereby with repressive chromatin marks, Dnmt1, and Hdac2 [76] (Figure 2).

The anterior pituitary is a postnatal mitotically active tissue and continues to grow until adulthood. Therefore, ELS could affect corticotroph cell numbers and confound results from ChIP experiments. Immunostaining for Acth revealed, however, no differences between control and ELS mice at PND 10 and three months [76]. At the same time, *in vivo* ChIP analysis showed reduced Mecp2 occupancy at the *Pomc* promoter in ELS mice, which was accompanied by a decreased recruitment of Dnmt1 [76]. Conversely, RNA-polymerase II binding was increased in ELS mice consistent with enhanced Pomc mRNA levels.

Avp can potentiate the effects of Crh on Acth secretion as result of the crosstalk between PKA and PKC signaling in rodent pituitary cells and a heterodimerization of the respective membrane-bound receptors [128]. On the other side, Mecp2 is phosphorylated and controlled by Ca^2+^ signaling and possibly also by cAMP [98]. This led us to ask whether ELS-induced hypersecretion of Avp drives Mecp2-S421 phosphorylation and derepression of *Pomc*. Contrary to this assumption, we could not detect Mecp2-S421 phospho-immunoreactivity in corticotroph cells at PND 10—neither in control nor in ELS mice—whereas weak signals in adulthood did not differ between treatment groups (unpublished observations).

Taken together, these results suggest to Mecp2 a role in the mediation of epigenetic programming of pituitary *Pomc* and extend its function to neuroendocrine tissues (Figure 2). However, modifications unrelated to S421 phosphorylation seem to regulate Mecp2 dissociation at the *Pomc* promoter following ELS in mice. In this respect, S80 phosphorylation has been suggested to control Mecp2 chromatin association at the *Pomc* promoter in mouse embryonic neurons and may provide an alternative route to experience-dependent programming [87]. Thus, further studies are necessary to assess the relevance of S80 phosphorylation and other posttranslational Mecp2 modifications for Pomc expression.

## 6. Conclusions

MECP2’s function in the brain has been originally inferred from mutational alterations manifesting as a severe neurodevelopmental disorder in humans. Studies in patients and mice suggest that the onset of syndromes coincides with the period during postnatal development when sensory-driven neuronal activity shapes cortical circuitry compatible with a role of MECP2 in synaptic functions. At the molecular level MECP2 recognizes and binds to epigenetic modifications such as DNA methylation and chromatin marks and recruits various protein complexes that can modify epigenetic states to regulate gene expression. Despite considerable efforts, MECP2 regulated gene networks remain poorly defined, both with respect to activation and repression. Interestingly, a number of recent studies suggest that neuronal activity-driven posttranslational modifications, in particular phosphorylation, are key to Mecp2’s function in rodents. These modifications occur in a temporospatial manner within specific neural circuitries important to cognitive function, reward, and mood. Psychostimulants and antidepressant drugs cause site-specific Mecp2 phosphorylation indicating that they may mediate part of their effects on mood and behavior through Mecp2-dependent pathways.

Epigenetic mechanisms are increasingly acknowledged for their role in dynamically mediating the effects of changing environments on the genetic blueprint. In this respect, DNA methylation has been recently shown to translate social experiences into lasting changes in gene expression underpinning distinct phenotypes. This form of “molecular plasticity” is thought to facilitate an organism’s capability to mount an adaptive response through integration of multilayered gene-environment interactions.

Early life adversity can elicit life-long increases in glucocorticoid secretion and alterations in the mechanisms that regulate HPA-axis activity. All of these events increase the risk for the development of stress-related disorders (including MDD, anxiety, and PTSD), a protracted course, and weaker response to therapy. Experience-dependent epigenetic programming of key drivers of the stress response in mice has been observed at different functional levels, including *Crh* in the hippocampus, *Avp* in the hypothalamus, and *Pomc* in the anterior pituitary. Neuronal activity-driven phosphorylation of Mecp2 (S421) seems to be a critical event in the initiation of epigenetic bookmarking in response to ELS. As a result, epigenetic programming of hypothalamic *Avp* and pituitary *Pomc* leads to sustained HPA-axis activity in response to ELS, while the effects on hippocampal *Crh* impair cognitive functions relevant to stress coping.

Overall, these studies assign to Mecp2 a more dynamic role in gene regulation than originally thought and a critical role in the mediation of early life trauma. While this review focused on Mecp2’s contribution to epigenetic programming of the HPA axis, other physiological systems malleable by early life experiences are likely to be affected as well. Lastly, preliminary data from mice suggest that the lasting behavioral effects of Mecp2 phosphorylation in response to early life adversity can be attenuated by timely environmental interventions. Further studies are necessary to translate these findings to humans in order to improve therapy of early life trauma.

## References

[1] Waddington C.H. (2014). The Strategy of the Genes. A Discussion of Some Aspects of Theoretical Biology.

[2] Russo V.E.A., Martienssen R.A., Riggs A.D. (1996). Epigenetic Mechanisms of Gene Regulation.

[3] Allis C.D., Jenuwein T., Reinberg D. (2007). Epigenetics.

[4] Ausió J., de Paz A.M., Esteller M. (2014). MeCP2: The long trip from a chromatin protein to neurological disorders. Trends Mol. Med..

[5] Chahrour M., Zoghbi H.Y. (2007). The story of rett syndrome: From clinic to neurobiology. Neuron.

[6] Murgatroyd C., Spengler D. (2012). Genetic variation in the epigenetic machinery and mental health. Curr. Psychiatry Rep..

[7] Bird A. (2002). DNA methylation patterns and epigenetic memory. Genes Dev..

[8] Zhang T.-Y., Meaney M.J. (2010). Epigenetics and the environmental regulation of the genome and its function. Annu. Rev. Psychol..

[9] Murgatroyd C., Wu Y., Bockmühl Y., Spengler D. (2010). Genes learn from stress: How infantile trauma programs us for depression. Epigenetics.

[10] Hoffmann A., Spengler D. (2014). DNA memories of early social life. Neuroscience.

[11] Goldberg A.D., Allis C.D., Bernstein E. (2007). Epigenetics: A landscape takes shape. Cell.

[12] Eccleston A., DeWitt N., Gunter C., Marte B., Nath D. (2007). Insight: Introduction Epigenetics. Nature.

[13] Tollefsbol T.O. (2010). Handbook of Epigenetics: The New Molecular and Medical Genetics.

[14] Armstrong L. (2013). Epigenetics.

[15] Lister R., Mukamel E.A., Nery J.R., Urich M., Puddifoot C.A., Johnson N.D., Lucero J., Huang Y., Dwork A.J., Schultz M.D. (2013). Global epigenomic reconfiguration during mammalian brain development. Science.

[16] Ooi S.K.T., O’Donnell A.H., Bestor T.H. (2009). Mammalian cytosine methylation at a glance. J. Cell Sci..

[17] Kareta M.S., Botello Z.M., Ennis J.J., Chou C., Chédin F. (2006). Reconstitution and mechanism of the stimulation of de Novo methylation by human DNMT3L. J. Biol. Chem..

[18] Saxonov S., Berg P., Brutlag D.L. (2006). A genome-wide analysis of CpG dinucleotides in the human genome distinguishes two distinct classes of promoters. Proc. Natl. Acad. Sci. USA.

[19] Gibney E.R., Nolan C.M. (2010). Epigenetics and gene expression. Heredity.

[20] Bell J.T., Pai A.A., Pickrell J.K., Gaffney D.J., Pique-Regi R., Degner J.F., Gilad Y., Pritchard J.K. (2011). DNA methylation patterns associate with genetic and gene expression variation in HapMap cell lines. Genome Biol..

[21] Thurman R.E., Rynes E., Humbert R., Vierstra J., Maurano M.T., Haugen E., Sheffield N.C., Stergachis A.B., Wang H., Vernot B. (2012). The accessible chromatin landscape of the human genome. Nature.

[22] Weber M., Hellmann I., Stadler M.B., Ramos L., Pääbo S., Rebhan M., Schübeler D. (2007). Distribution, silencing potential and evolutionary impact of promoter DNA methylation in the human genome. Nat. Genet..

[23] Zemach A., McDaniel I.E., Silva P., Zilberman D. (2010). Genome-Wide Evolutionary Analysis of Eukaryotic DNA Methylation. Science.

[24] Feng S., Cokus S.J., Zhang X., Chen P.-Y., Bostick M., Goll M.G., Hetzel J., Jain J., Strauss S.H., Halpern M.E. (2010). Conservation and divergence of methylation patterning in plants and animals. Proc. Natl. Acad. Sci. USA.

[25] Maunakea A.K., Nagarajan R.P., Bilenky M., Ballinger T.J., D’Souza C., Fouse S.D., Johnson B.E., Hong C., Nielsen C., Zhao Y. (2010). Conserved role of intragenic DNA methylation in regulating alternative promoters. Nature.

[26] Gibbs J.R., van der Brug M.P., Hernandez D.G., Traynor B.J., Nalls M.A., Lai S.-L., Arepalli S., Dillman A., Rafferty I.P., Troncoso J. (2010). Abundant quantitative trait loci exist for DNA methylation and gene expression in human brain. PLoS Genet..

[27] Gutierrez-Arcelus M., Lappalainen T., Montgomery S.B., Buil A., Ongen H., Yurovsky A., Bryois J., Giger T., Romano L., Planchon A. (2013). Passive and active DNA methylation and the interplay with genetic variation in gene regulation. eLife.

[28] Wu H., Zhang Y. (2014). Reversing DNA methylation: Mechanisms, genomics, and Biological functions. Cell.

[29] Tahiliani M., Koh K.P., Shen Y., Pastor W.A., Bandukwala H., Brudno Y., Agarwal S., Iyer L.M., Liu D.R., Aravind L. (2009). Conversion of 5-methylcytosine to 5-hydroxymethylcytosine in mammalian DNA by MLL partner TET1. Science.

[30] Ito S., D’Alessio A.C., Taranova O.V., Hong K., Sowers L.C., Zhang Y. (2010). Role of Tet proteins in 5mC to 5hmC conversion, ES-cell self-renewal and inner cell mass specification. Nature.

[31] Ito S., Shen L., Dai Q., Wu S.C., Collins L.B., Swenberg J.A., He C., Zhang Y. (2011). Tet proteins can convert 5-methylcytosine to 5-formylcytosine and 5-carboxylcytosine. Science.

[32] He Y.-F., Li B.-Z., Li Z., Liu P., Wang Y., Tang Q., Ding J., Jia Y., Chen Z., Li L. (2011). Tet-mediated formation of 5-carboxylcytosine and its excision by TDG in mammalian DNA. Science.

[33] Cortellino S., Xu J., Sannai M., Moore R., Caretti E., Cigliano A., le Coz M., Devarajan K., Wessels A., Soprano D. (2011). Thymine DNA glycosylase is essential for active DNA demethylation by linked deamination-base excision repair. Cell.

[34] Guo J.U., Su Y., Zhong C., Ming G., Song H. (2011). Emerging roles of TET proteins and 5-hydroxymethylcytosines in active DNA demethylation and beyond. Cell Cycle.

[35] Maiti A., Drohat A.C. (2011). Thymine DNA Glycosylase Can Rapidly Excise 5-formylcytosine and 5-carboxylcytosine potential implications for active demethylation of CpG sites. J. Biol. Chem..

[36] Spruijt C.G., Gnerlich F., Smits A.H., Pfaffeneder T., Jansen P.W.T.C., Bauer C., Münzel M., Wagner M., Müller M., Khan F. (2013). Dynamic readers for 5-(hydroxy)methylcytosine and its oxidized derivatives. Cell.

[37] Li B., Carey M., Workman J.L. (2007). The role of chromatin during transcription. Cell.

[38] Misteli T. (2007). Beyond the sequence: Cellular organization of genome function. Cell.

[39] Cairns B.R. (2007). Chromatin remodeling: Insights and intrigue from single-molecule studies. Nat. Struct. Mol. Biol..

[40] Berger S.L. (2007). The complex language of chromatin regulation during transcription. Nature.

[41] Bernstein B.E., Meissner A., Lander E.S. (2007). The mammalian epigenome. Cell.

[42] Hendrich B., Bird A. (1998). Identification and characterization of a family of mammalian Methyl-CpG binding proteins. Mol. Cell. Biol..

[43] Dani V.S., Chang Q., Maffei A., Turrigiano G.G., Jaenisch R., Nelson S.B. (2005). Reduced cortical activity due to a shift in the balance between excitation and inhibition in a mouse model of Rett Syndrome. Proc. Natl. Acad. Sci. USA.

[44] Chao H.-T., Zoghbi H.Y., Rosenmund C. (2007). MeCP2 controls excitatory synaptic strength by regulating glutamatergic synapse number. Neuron.

[45] Tropea D., Giacometti E., Wilson N.R., Beard C., McCurry C., Fu D.D., Flannery R., Jaenisch R., Sur M. (2009). Partial reversal of Rett Syndrome-like symptoms in MeCP2 mutant mice. Proc. Natl. Acad. Sci. USA.

[46] Adkins N.L., Georgel P.T. (2010). MeCP2: structure and function. Biochem. Cell. Biol..

[47] Pelka G.J., Watson C.M., Christodoulou J., Tam P.P.L. (2005). Distinct expression profiles of Mecp2 transcripts with different lengths of 3'UTR in the brain and visceral organs during mouse development. Genomics.

[48] Shahbazian M.D., Antalffy B., Armstrong D.L., Zoghbi H.Y. (2002). Insight into Rett syndrome: MeCP2 levels display tissue- and cell-specific differences and correlate with neuronal maturation. Hum. Mol. Genet..

[49] Na E.S., Monteggia L.M. (2011). The role of MeCP2 in CNS development and function. Horm. Behav..

[50] Ho K.L., McNae I.W., Schmiedeberg L., Klose R.J., Bird A.P., Walkinshaw M.D. (2008). MeCP2 binding to DNA depends upon hydration at methyl-CpG. Mol. Cell.

[51] Klose R.J., Sarraf S.A., Schmiedeberg L., McDermott S.M., Stancheva I., Bird A.P. (2005). DNA binding selectivity of MeCP2 due to a requirement for A/T sequences adjacent to methyl-CpG. Mol. Cell.

[52] Jones P.L., Jan Veenstra G.C., Wade P.A., Vermaak D., Kass S.U., Landsberger N., Strouboulis J., Wolffe A.P. (1998). Methylated DNA and MeCP2 recruit histone deacetylase to repress transcription. Nat. Genet..

[53] Nan X., Campoy F.J., Bird A. (1997). MeCP2 is a transcriptional repressor with abundant binding sites in genomic chromatin. Cell.

[54] Nikitina T., Shi X., Ghosh R.P., Horowitz-Scherer R.A., Hansen J.C., Woodcock C.L. (2007). Multiple modes of interaction between the methylated DNA binding protein MeCP2 and chromatin. Mol. Cell. Biol..

[55] Nan X., Hou J., Maclean A., Nasir J., Lafuente M.J., Shu X., Kriaucionis S., Bird A. (2007). Interaction between chromatin proteins MECP2 and ATRX is disrupted by mutations that cause inherited mental retardation. Proc. Natl. Acad. Sci. USA.

[56] Kimura H., Shiota K. (2003). Methyl-CpG-binding protein, MeCP2, is a target molecule for maintenance DNA methyltransferase, Dnmt1. J. Biol. Chem..

[57] Fuks F., Hurd P.J., Wolf D., Nan X., Bird A.P., Kouzarides T. (2003). The Methyl-CpG-binding protein MeCP2 links DNA methylation to histone methylation. J. Biol. Chem..

[58] Kokura K., Kaul S.C., Wadhwa R., Nomura T., Khan M.M., Shinagawa T., Yasukawa T., Colmenares C., Ishii S. (2001). The ski protein family is required for MeCP2-mediated transcriptional repression. J. Biol. Chem..

[59] Jeffery L., Nakielny S. (2004). Components of the DNA methylation system of chromatin control are RNA-binding proteins. J. Biol. Chem..

[60] Skene P.J., Illingworth R.S., Webb S., Kerr A.R.W., James K.D., Turner D.J., Andrews R., Bird A.P. (2010). Neuronal MeCP2 is expressed at near histone-octamer levels and globally alters the chromatin state. Mol. Cell.

[61] Georgel P.T., Horowitz-Scherer R.A., Adkins N., Woodcock C.L., Wade P.A., Hansen J.C. (2003). Chromatin compaction by human MeCP2 assembly of novel secondary chromatin structures in the absence of DNA methylation. J. Biol. Chem..

[62] Yasui D.H., Peddada S., Bieda M.C., Vallero R.O., Hogart A., Nagarajan R.P., Thatcher K.N., Farnham P.J., LaSalle J.M. (2007). Integrated epigenomic analyses of neuronal MeCP2 reveal a role for long-range interaction with active genes. Proc. Natl. Acad. Sci. USA.

[63] Harikrishnan K.N., Bayles R., Ciccotosto G.D., Maxwell S., Cappai R., Pelka G.J., Tam P.P.L., Christodoulou J., El-Osta A. (2010). Alleviating transcriptional inhibition of the norepinephrine Slc6a2 transporter gene in depolarized neurons. J. Neurosci..

[64] Kernohan K.D., Jiang Y., Tremblay D.C., Bonvissuto A.C., Eubanks J.H., Mann M.R.W., Bérubé N.G. (2010). ATRX Partners with cohesin and MeCP2 and contributes to developmental silencing of imprinted genes in the brain. Dev. Cell.

[65] Tudor M., Akbarian S., Chen R.Z., Jaenisch R. (2002). Transcriptional profiling of a mouse model for Rett syndrome reveals subtle transcriptional changes in the brain. Proc. Natl. Acad. Sci. USA.

[66] Guy J., Cheval H., Selfridge J., Bird A. (2011). The role of MeCP2 in the brain. Annu. Rev. Cell Dev. Biol..

[67] Zachariah R.M., Rastegar M. (2012). Linking epigenetics to human disease and Rett syndrome: The emerging novel and challenging concepts in MeCP2 research. Neural Plast..

[68] Chahrour M., Jung S.Y., Shaw C., Zhou X., Wong S.T.C., Qin J., Zoghbi H.Y. (2008). MeCP2, a key contributor to neurological disease, activates and represses transcription. Science.

[69] Ben-Shachar S., Chahrour M., Thaller C., Shaw C.A., Zoghbi H.Y. (2009). Mouse models of MeCP2 disorders share gene expression changes in the cerebellum and hypothalamus. Hum. Mol. Genet..

[70] Jordan C., Francke U. (2006). Ube3a expression is not altered in Mecp2 mutant mice. Hum. Mol. Genet..

[71] Kriaucionis S., Paterson A., Curtis J., Guy J., MacLeod N., Bird A. (2006). Gene expression analysis exposes mitochondrial abnormalities in a mouse model of rett syndrome. Mol. Cell. Biol..

[72] Nuber U.A., Kriaucionis S., Roloff T.C., Guy J., Selfridge J., Steinhoff C., Schulz R., Lipkowitz B., Ropers H.H., Holmes M.C. (2005). Up-regulation of glucocorticoid-regulated genes in a mouse model of Rett syndrome. Hum. Mol. Genet..

[73] Lang F., Strutz-Seebohm N., Seebohm G., Lang U.E. (2010). Significance of SGK1 in the regulation of neuronal function. J. Physiol..

[74] Storer C.L., Dickey C.A., Galigniana M.D., Rein T., Cox M.B. (2011). FKBP51 and FKBP52 in signaling and disease. Trends Endocrinol. Metab..

[75] Murgatroyd C., Spengler D. (2011). Epigenetic programming of the HPA axis: Early life decides. Stress.

[76] Wu Y., Patchev A.V., Daniel G., Almeida O.F.X., Spengler D. (2014). Early-life stress reduces DNA methylation of the Pomc gene in male mice. Endocrinology.

[77] McGill B.E., Bundle S.F., Yaylaoglu M.B., Carson J.P., Thaller C., Zoghbi H.Y. (2006). Enhanced anxiety and stress-induced corticosterone release are associated with increased Crh expression in a mouse model of Rett syndrome. Proc. Natl. Acad. Sci. USA.

[78] Müller M.B., Uhr M., Holsboer F., Keck M.E. (2004). Hypothalamic-pituitary-adrenocortical system and mood disorders: Highlights from mutant mice. Neuroendocrinology.

[79] Zhou Z., Hong E.J., Cohen S., Zhao W., Ho H.H., Schmidt L., Chen W.G., Lin Y., Savner E., Griffith E.C. (2006). Brain-specific phosphorylation of MeCP2 regulates activity-dependent Bdnf transcription, dendritic growth, and spine maturation. Neuron.

[80] Samaco R.C., Mandel-Brehm C., McGraw C.M., Shaw C.A., McGill B.E., Zoghbi H.Y. (2012). Crh and Oprm1 mediate anxiety-related behavior and social approach in a mouse model of MECP2 duplication syndrome. Nat. Genet..

[81] Ramocki M.B., Peters S.U., Tavyev Y.J., Zhang F., Carvalho C.M.B., Schaaf C.P., Richman R., Fang P., Glaze D.G., Lupski J.R. (2009). Autism and other neuropsychiatric symptoms are prevalent in individuals with MeCP2 duplication syndrome. Ann. Neurol..

[82] Li W., Pozzo-Miller L. (2014). BDNF deregulation in Rett syndrome. Neuropharmacology.

[83] Bellini E., Pavesi G., Barbiero I., Bergo A., Chandola C., Nawaz M.S., Rusconi L., Stefanelli G., Strollo M., Valente M.M. (2014). MeCP2 post-translational modifications: A mechanism to control its involvement in synaptic plasticity and homeostasis?. Front. Cell. Neurosci..

[84] Li H., Chang Q. (2014). Regulation and function of stimulus-induced phosphorylation of MeCP2. Front. Biol..

[85] Martinowich K., Hattori D., Wu H., Fouse S., He F., Hu Y., Fan G., Sun Y.E. (2003). DNA methylation-related chromatin remodeling in activity-dependent Bdnf gene regulation. Science.

[86] Chen W.G., Chang Q., Lin Y., Meissner A., West A.E., Griffith E.C., Jaenisch R., Greenberg M.E. (2003). Derepression of BDNF transcription involves calcium-dependent phosphorylation of MeCP2. Science.

[87] Tao J., Hu K., Chang Q., Wu H., Sherman N.E., Martinowich K., Klose R.J., Schanen C., Jaenisch R., Wang W. (2009). Phosphorylation of MeCP2 at Serine 80 regulates its chromatin association and neurological function. Proc. Natl. Acad. Sci. USA.

[88] Bracaglia G., Conca B., Bergo A., Rusconi L., Zhou Z., Greenberg M.E., Landsberger N., Soddu S., Kilstrup‐Nielsen C. (2009). Methyl‐CpG‐binding protein 2 is phosphorylated by homeodomain‐interacting protein kinase 2 and contributes to apoptosis. EMBO Rep..

[89] Gonzales M.L., Adams S., Dunaway K.W., LaSalle J.M. (2012). Phosphorylation of distinct sites in MeCP2 modifies cofactor associations and the dynamics of transcriptional regulation. Mol. Cell. Biol..

[90] Cohen S., Gabel H.W., Hemberg M., Hutchinson A.N., Sadacca L.A., Ebert D.H., Harmin D.A., Greenberg R.S., Verdine V.K., Zhou Z. (2011). Genome-wide activity-dependent MeCP2 phosphorylation regulates nervous system development and function. Neuron.

[91] Li H., Zhong X., Chau K.F., Williams E.C., Chang Q. (2011). Loss of activity-induced phosphorylation of MeCP2 enhances synaptogenesis, LTP and spatial memory. Nat. Neurosci..

[92] Nestler E.J. (2001). Molecular basis of long-term plasticity underlying addiction. Nat. Rev. Neurosci..

[93] Hyman S.E., Malenka R.C., Nestler E.J. (2006). Neural mechanisms of addiction: The role of reward-related learning and memory. Annu. Rev. Neurosci..

[94] Cassel S., Carouge D., Gensburger C., Anglard P., Burgun C., Dietrich J.-B., Aunis D., Zwiller J. (2006). Fluoxetine and cocaine induce the epigenetic factors MeCP2 and MBD1 in adult rat brain. Mol. Pharmacol..

[95] Deng J.V., Rodriguiz R.M., Hutchinson A.N., Kim I.-H., Wetsel W.C., West A.E. (2010). MeCP2 in the nucleus accumbens contributes to neural and behavioral responses to psychostimulants. Nat. Neurosci..

[96] Mao L.-M., Horton E., Guo M.-L., Xue B., Jin D.-Z., Fibuch E.E., Wang J.Q. (2011). Cocaine increases phosphorylation of MeCP2 in the rat striatum *in vivo*: A differential role of NMDA receptors. Neurochem. Int..

[97] Deng J.V., Wan Y., Wang X., Cohen S., Wetsel W.C., Greenberg M.E., Kenny P.J., Calakos N., West A.E. (2014). MeCP2 phosphorylation limits psychostimulant-induced behavioral and neuronal plasticity. J. Neurosci..

[98] Hutchinson A.N., Deng J.V., Aryal D.K., Wetsel W.C., West A.E. (2012). Differential regulation of MeCP2 phosphorylation in the CNS by dopamine and serotonin. Neuropsychopharmacology.

[99] Schloesser R.J., Martinowich K., Manji H.K. (2012). Mood-stabilizing drugs: Mechanisms of action. Trends Neurosci..

[100] Hikosaka O. (2010). The habenula: from stress evasion to value-based decision-making. Nat. Rev. Neurosci..

[101] Green J., McLaughlin K.A., Berglund P.A. (2010). Childhood adversities and adult psychiatric disorders in the national comorbidity survey replication i: Associations with first onset of DSM-IV disorders. Arch. Gen. Psychiatry.

[102] U.S. Department of Health and Human Services; Administration for Children and Families; Administration on Children, Youth and Families. Children’s Bureau Child. Maltreatment 2009.

[103] U.S. Department of Health and Human Services; Administration for Children and Families; Administration on Children, Youth and Families; Children’s Bureau Child. Maltreatment 2010.

[104] Briere J., Berliner L., Bulkley J.A., Jenny C., Reid T., American Professional Society on the Abuse of Children (1996). The APSAC Handbook on Child Maltreatment.

[105] Olson S., Institute of Medicine (U.S.), National Research Council (U.S.) (2012). From Neurons to Neighborhoods: An Update: Workshop Summary.

[106] England M.J., Sim L.J., National Research Council (U.S.), Institute of Medicine (U.S.) (2009). Depression in Parents, Parenting, and Children: Opportunities to Improve Identification, Treatment, and Prevention.

[107] Edwards V.J., Holden G.W., Felitti V.J., Anda R.F. (2003). Relationship between multiple forms of childhood maltreatment and adult mental health in community respondents: results from the adverse childhood experiences study. Am. J. Psychiatry.

[108] Felitti V.J., Anda R.F., Nordenberg D., Williamson D.F., Spitz A.M., EdwardS V., Koss M.P., Marks J.S. (1998). Relationship of childhood abuse and household dysfunction to many of the leading causes of death in adults: The adverse childhood experiences (ACE) study. Am. J. Prev. Med..

[109] Chapman D.P., Whitfield C.L., Felitti V.J., Dube S.R., Edwards V.J., Anda R.F. (2004). Adverse childhood experiences and the risk of depressive disorders in adulthood. J. Affect. Disord..

[110] Dube S.R., Anda R.F., Felitti V.J., Chapman D.P., Williamson D.F., Giles W.H. (2001). Childhood abuse, household dysfunction, and the risk of attempted suicide throughout the life span: Findings from the adverse childhood experiences study. JAMA.

[111] Raabe F.J., Spengler D. (2013). Epigenetic risk factors in PTSD and depression. Front. Mol. Psychiatry.

[112] Lupien S.J., McEwen B.S., Gunnar M.R., Heim C. (2009). Effects of stress throughout the lifespan on the brain, behaviour and cognition. Nat. Rev. Neurosci..

[113] McCrory E., De Brito S.A., Viding E. (2010). Research Review: The neurobiology and genetics of maltreatment and adversity. J. Child. Psychol. Psychiatry.

[114] Lupien S.J., Fiocco A., Wan N., Maheu F., Lord C., Schramek T., Tu M.T. (2005). Stress hormones and human memory function across the lifespan. Psychoneuroendocrinology.

[115] Hoffmann A., Spengler D. (2012). The lasting legacy of social stress on the epigenome of the hypothalamic–pituitary–adrenal axis. Epigenomics.

[116] Patchev A.V., Rodrigues A.J., Sousa N., Spengler D., Almeida O.F.X. (2014). The future is now: early life events preset adult behaviour. Acta. Physiol..

[117] Fink G. (2007). Encyclopedia of Stress.

[118] Murgatroyd C., Patchev A.V., Wu Y., Micale V., Bockmühl Y., Fischer D., Holsboer F., Wotjak C.T., Almeida O.F.X., Spengler D. (2009). Dynamic DNA methylation programs persistent adverse effects of early-life stress. Nat. Neurosci..

[119] Goncharova N.D. (2013). Stress responsiveness of the hypothalamic-pituitary-adrenal axis: Age-related features of the vasopressinergic regulation. Front. Endocrinol..

[120] Makara G.B., Varga J., Barna I., Pintér O., Klausz B., Zelena D. (2012). The vasopressin-deficient brattleboro rat: Lessons for the hypothalamo-pituitary-adrenal axis regulation. Cell. Mol. Neurobiol..

[121] Schmidt M.V., Sterlemann V., Wagner K., Niederleitner B., Ganea K., Liebl C., Deussing J.M., Berger S., Schütz G., Holsboer F. (2009). Postnatal glucocorticoid excess due to pituitary glucocorticoid receptor deficiency: Differential short- and long-term consequences. Endocrinology.

[122] Murgatroyd C., Spengler D. (2014). Polycomb binding precedes early-life stress responsive DNA methylation at the avp enhancer. PLOS ONE.

[123] Menger Y., Bettscheider M., Murgatroyd C., Spengler D. (2010). Sex differences in brain epigenetics. Epigenomics.

[124] Chen Y., Bender R.A., Frotscher M., Baram T.Z. (2001). Novel and transient populations of corticotropin-releasing hormone-expressing neurons in developing hippocampus suggest unique functional roles: A quantitative spatiotemporal analysis. J. Neurosci..

[125] Chen Y., Andres A., Frotscher M., Baram T.Z. (2012). Tuning synaptic transmission in the hippocampus by stress: the CRH system. Front. Cell. Neurosci..

[126] Maras P.M., Baram T.Z. (2012). Sculpting the hippocampus from within: stress, spines, and CRH. Trends Neurosci..

[127] Wang A., Nie W., Li H., Hou Y., Yu Z., Fan Q., Sun R. (2014). Epigenetic upregulation of corticotrophin-releasing hormone mediates postnatal maternal separation-induced memory deficiency. PLOS ONE.

[128] Murat B., Devost D., Andrés M., Mion J., Boulay V., Corbani M., Zingg H.H., Guillon G. (2012). V1b and CRHR1 receptor heterodimerization mediates synergistic biological actions of vasopressin and CRH. Mol. Endocrinol..

